# The feasibility and acceptability of conducting a randomised controlled trial evaluating a healthcare professional-supported, self-management intervention for people suffering from fatigue after critical illness

**DOI:** 10.1186/s40814-026-01798-7

**Published:** 2026-03-14

**Authors:** Yijun Yu, Sophie Eleanor Brown, Akshay Shah, Wladyslawa Czuber-Dochan, Suzanne Bench, Clare Martin, Georgia Cook, David McWilliams, Emma Hedley, Najib Rahman, Rebecca Langley, Louise Stayt

**Affiliations:** 1 Oxford Institute of Applied Health Research (OxInAHR), Oxford Brookes University, Headington Campus, Oxford, OX3 0BP UK; 2 Nuffield Department of Clinical Neurosciences, University of Oxford, Oxford, UK; 3 Department of Anaesthesia, Hammersmith Hospital, Imperial College Healthcare NHS Trust, London, UK; 4 Florence Nightingale Faculty of Nursing, Midwifery and Palliative Care, King’s College London, London, UK; 5 College of Healthcare Science, London South Bank University, London, UK; 6 Guys & St Thomas’ NHS Foundation Trust, London, UK; 7 School of Engineering, Computing and Mathematics, Oxford Brookes University, Oxford, UK; 8 Centre for Care Excellence, Coventry University, Coventry, UK; 9 University Hospitals Coventry & Warwickshire, Coventry, UK; 10 Oxford Respiratory Trials Unit, Nuffield Department of Medicine, University of Oxford, Oxford, UK; 11 Oxford NIHR Biomedical Research Centre, Oxford, UK; 12 Chinese Academy of Medicine Oxford Institute, Oxford, UK; 13 Patient co-applicant, Oxford, UK

**Keywords:** Critical illness, Intensive care unit, Fatigue, Self-management, Feasibility

## Abstract

**Background:**

Fatigue is a common problem that significantly affects intensive care unit (ICU) survivors’ physical, psychological, and social functioning. ICU survivors often experience a loss of self-worth and identity, struggle to return to their normal roles, and face ongoing challenges with cognitive and emotional recovery. Despite its profound impact, there are limited rehabilitation interventions targeting this population. This research aims to evaluate the acceptability of implementing the Fatigue After CriTical illness (FACT) self-management intervention into usual care for patients experiencing fatigue after critical illness and the feasibility of the intervention for a future clinical trial.

**Methods:**

This is a multicentre, open-label, feasibility randomised controlled trial (RCT) with an embedded qualitative evaluation, conducted across. Seventy participants recruited from three United Kingdom (UK) National Health Service (NHS) Trusts will be randomised either to the FACT self-management intervention following hospital discharge, which focuses on fatigue management, goal setting, and personal action planning, in addition to usual care, or to a control group receiving usual care alone. The intervention will be accessible for six months and will include a 30-min phone or video call with an ICU follow-up healthcare professional (HCP) in the third month. HCPs will attend an online training session and follow a protocol to guide patients in goal setting. Outcome assessments will occur at baseline, three months, and six months post-randomisation to evaluate feasibility and acceptability. Semi-structured interviews with patients and HCPs will explore their experiences and acceptability outcomes at 6 months.

**Discussion:**

This study aims to provide insights into the feasibility and acceptability of the FACT intervention, with the goal of improving fatigue management among survivors of critical illness. Preliminary findings will inform the design of a larger-scale RCT to evaluate its effectiveness in enhancing recovery from critical illness in patients who are experiencing fatigue.

**Trial registration:**

Study ID: ISRCTN1381359. Date registered: 01/04/2025.

**Supplementary Information:**

The online version contains supplementary material available at 10.1186/s40814-026-01798-7.

## Background

Fatigue is a distressing and debilitating symptom experienced by people who are recovering from critical illness and is described as an overwhelming mental and physical exhaustion [1, 2, 29]. As highlighted in the context of post-intensive care syndrome, intensive care unit (ICU) survivors, similar to many other patient populations, often face clusters of symptoms rather than isolated impairments. These symptoms affect multiple functional domains [8] and lead to diminished mental and physical capacities, impacting various aspects of daily life [1, 8]. This can include a loss of identity and self-esteem, sleep disturbances, and cognitive fatigue, which negatively influence concentration, thought processes, and memory [12, 17]. Fatigue has a detrimental impact on physical and psychological well-being and health-related quality of life (HRQoL) in people recovering from critical illness, and is one of the three most significant problems described by people recovering from critical illness, along with a lack of physical strength and decreased walking distance [28]. Our systematic review found that up to 81% of people report symptoms of fatigue 4 months after ICU discharge, with 50% (equating to 82,000 per year in the UK) reporting persistent fatigue at 1 year [2]. 

People recovering from critical illness often rely heavily on the support of others and may be unable to carry out their usual family, social, and work roles, leading to reduced self-worth or a loss of identity [1, 2]. This can be compounded by a lack of understanding and empathy from healthcare professionals (HCPs), employers, and family members [1, 2, 26]. People who have experienced critical illness often report requiring assistance with activities of daily living (ADLs) (e.g. toileting, hygiene) many months after hospital discharge [1, 34]. People in the first year after ICU discharge also have multiple encounters with primary care physicians [25] and Schmidt et al. [33] found that many require long-term aftercare. Helping people manage their fatigue after critical illness has the potential to increase engagement with rehabilitation, improve physical performance, promote social (re)integration, and improve overall HRQoL.

Despite its prevalence, fatigue remains under-recognised in the current literature. Our systematic review found that there were no existing interventions to support rehabilitation specifically for people with fatigue after critical illness [2]. However, our umbrella review of self-management interventions for fatigue secondary to any acute or long-term condition found that these interventions had positive effects on patients, particularly when supported by HCPs [6]. Survivors require skill-building, interprofessional support, and tailored strategies to break the cycle of fatigue and inactivity [18, 20].

Although definitions of self-management vary, it generally refers to self-directed activities, where people take a leading role in managing their symptoms. The primary goal is often to improve coping and illness management, rather than achieving objective symptom improvement—though this may also occur [6]. Common self-management activities include accessing educational materials, symptom tracking, goal setting, and monitoring progress [14, 24]. Goal setting has proven effective in self-management strategies for energy conservation treatment [3] as it helps people to organise themselves in a way that maximises engagement while minimising energy depletion [20].

To address the need for targeted rehabilitation, we co-produced, with ICU survivors and carers, a theory-based, HCP-supported self-management intervention for fatigue after critical illness, known as FACT (managing Fatigue After CriTical illness), and tested it with service users. Three workshops were conducted with people experiencing fatigue after critical illness, along with family members and HCPs, to co-develop the first draft of the FACT intervention, which was subsequently designed in online and electronic formats. User testing (*n* = 4) found that the intervention was well received. Participants valued the intervention, reporting that it helped them to manage their fatigue and prioritise daily activities [7].

## Aims and objectives

This study aims to determine the feasibility and acceptability of conducting a randomised controlled trial (RCT) of the FACT intervention. The objectives are:

- Primary objective: To assess the willingness of people affected by fatigue after critical illness to participate in an RCT evaluating an HCP-supported self-management intervention for fatigue after critical illness.
- Secondary objectives:
  1. To estimate recruitment, retention, and the variability of outcome measures to inform sample size calculations and choice of outcome measures for a future effectiveness trial.
  2. To evaluate the acceptability and fidelity of the intervention by assessing adherence, estimated frequency of visiting the website, and patterns of use.
  3. To evaluate HCPs’ perceptions and experiences of delivering support for this intervention.

## Materials and methods

The study protocol is reported using the Standard Protocol Items: Recommendations for Interventions (SPIRIT) statement guidelines [10, 11] and the publication associated with the trial’s findings will be reported in accordance with the CONSORT extension for randomised pilot and feasibility trials [16]. A SPIRIT checklist is provided in Additional file 1.

### Study design

The study is a multicentre, open-label, feasibility RCT, with a qualitative evaluation of acceptability, conducted across ICUs at three UK NHS Trusts. A total of 70 participants will be randomised in a 1:1 ratio by sealed envelopes to receive the FACT intervention in addition to usual care or usual care alone. The intervention will be made available to the patients on hospital discharge and be accessible for 6 months. Participants will have a 30-min supportive phone/video call with an HCP from the ICU follow-up team at each site for three months. HCPs will attend an online training event and follow a defined protocol to facilitate goal-setting and personal action-planning. This study is an open-label trial; therefore, there will be no blinding to the participants or the trial management team.

**Fig. 1:**
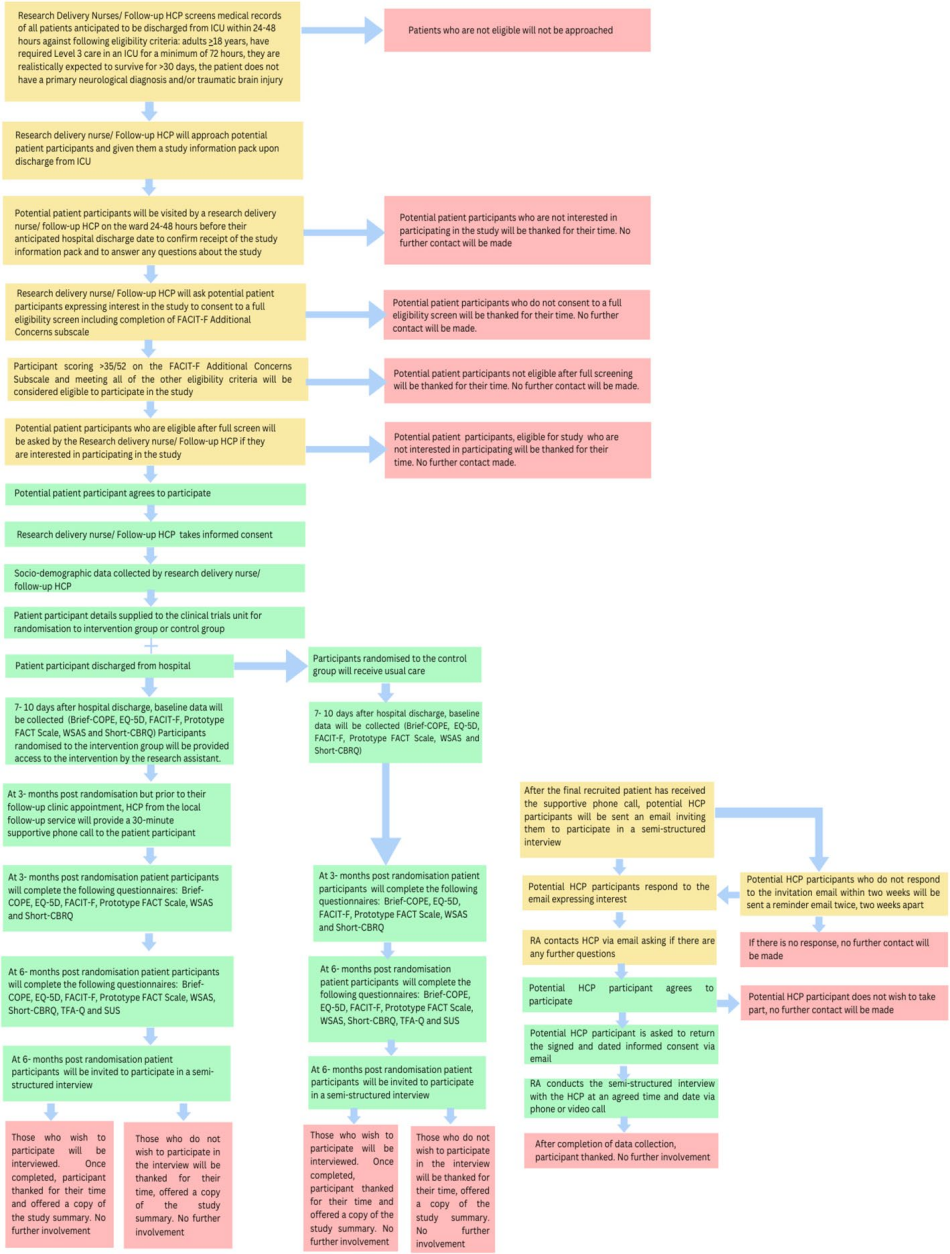
Study flow chart

All participating research sites offer inpatient ICU follow-up services. Patients who were in the ICU for more than 72 h are offered outpatient services at three months after hospital discharge, where physical and psychological health outcomes are evaluated. Where necessary, appropriate referrals and treatment plans are made. In the control group, patients (*n* = 35) will receive standard care, which includes routine follow-up services at their respective sites. Standard care typically involves a review of the patient’s recovery status without any additional interventions provided by the study. Additionally, patients may access generic information about recovery after critical illness, which is available on a variety of websites, including resources addressing fatigue. A flow chart of the study is presented in Fig. 1.

### Study setting

Researchers in the Faculty of Health, Science and Technology, Oxford Brookes University, will coordinate the study. The intervention is designed as a website and will be self-managed by participants at home, with HCP support taking place via phone/video calls. Usual care will involve ICU follow-up services to patients at the three sites.

### Participants

#### Inclusion criteria

Patient participants will be eligible for inclusion if they (i) are adults ≥ 18 years; (ii) have been in an ICU for a minimum of 72 h; (iii) have required Level 3 care (failure of two or more organs and/or requiring advanced respiratory care) [21] during their ICU stay; (iv) self-report symptoms of fatigue at the point of discharge (± 7 days) from hospital (≤ 34/52) on the validated Functional Assessment of Chronic Illness Therapy-Fatigue (FACIT-F) additional concerns subscale [37]; (v) are ready for discharge from hospital (± 7 days); and (vi) are willing and able to comply with the study protocol requirements and procedures.

#### Exclusion criteria

Prospective patient participants will be excluded if they (i) are expected to survive for ≤ 30 days according to their clinical team and (ii) have a neurological diagnosis occurring as a cause or result of the patient ICU admission (e.g. stroke, traumatic brain injury) or any neurodegenerative condition (e.g. multiple sclerosis, amyotrophic lateral sclerosis) as these specialist groups have different clinical and biological characteristics.

### Sample size

Based on our case-mix data, we have a target sample of 70 participants with 1:1 randomised to the intervention and control arms. As this is a feasibility trial, a power calculation has not been conducted. Seventy participants would provide adequate feasibility data [35] and allow timely completion of the study. Anticipating a participant retention rate of 80%, we estimated that a target sample of 70 would allow us to calculate retention with a margin of error of approximately 18% with a 95% confidence level. This sample size was deemed sufficient to inform the future RCT and to provide adequate analysis of the study’s progression criteria.

HCPs involved in the delivery of the intervention at each site will be purposely sampled from each site. It is anticipated that 2–5 HCPs from each site will be involved in the intervention delivery. Eligible HCPs will include those registered with a professional body (Nursing and Midwifery Council, General Medical Council, Health, and Care Professionals Council) and who are directly involved in the delivery of the intervention for this study.

### Recruitment

This study will recruit patients from ICUs from three NHS Trusts: Oxford University Hospital Foundation Trust (OUHFT), Guy’s and St. Thomas’ NHS Foundation Trust (GSTT), and University Hospitals Coventry and Warwickshire NHS Trust (UHCWT). These trusts cover geographically diverse patient populations, including a range of case complexity, ethnic backgrounds, and levels of social deprivation.

### Intervention

The FACT self-management intervention is based on the common-sense model of self-regulation [23] and consists of four structured modules covering aspects of fatigue management, including (i) About fatigue; (ii) Managing your energy with the 5 Ps (Priorities, Pacing, Planning, Permission, Position); (iii) Strategies for everyday life; and (iv) Goal setting and making plans. The intervention is separated into 16 sections and includes information in the form of both videos and text, activities, as well as patient stories. It is available via a website with all information and activities available to download and accessible in multi-formats (infographics, videos, printable and editable documents). A printed booklet will also be available according to patient preferences.

Patients will be encouraged but not required to move through the topics in the order presented and will be able to use the intervention at their own pace, repeating any topics if desired. As described in “Trial Design”, participants will receive a 30-min supportive phone or video call with an HCP from the ICU follow-up team at three months post-discharge. During this call, the HCP will assist with goal setting and action planning. To ensure consistency and quality of delivery, HCPs will attend an online training session and follow a defined protocol. In addition, a manual for research delivery nurses has been developed, which includes a clear timeline and action checklist to guide screening, recruitment, and participant follow-up. To support the delivery of the intervention, supportive call materials have also been prepared, including a supportive phone call structure and example scripts to help HCPs conduct the phone calls effectively and maintain fidelity to the intervention framework.

The control arm will receive usual care across all the research sites; existing inpatient ICU follow-up services are offered. These include inpatient ICU follow-up services, and patients who were in ICU for more than 72 h are offered outpatient services three months after hospital discharge, where physical and psychological health outcomes are evaluated. Where necessary, appropriate referrals and treatment plans are made. In addition, patients may also have access to generic information about recovery after critical illness available on a variety of websites (ICU Steps, criticalcarerecovery.com), which includes information about fatigue.

This trial is funded for a period of 20 months and commences in October 2025. It is anticipated that recruitment will be completed by the end of 2025. Final assessments, data analysis, reporting and write-up of results/findings will be completed by May 2026.

### Data management

All study data will be entered into REDCap, a secure, validated electronic data capture system. REDCap ensures data accuracy and integrity through a range of built-in checks, automated error detection, and logging of data modifications. The system is programmed to only allow data entries within predefined value ranges, minimising the potential for errors. Participants will be identified within REDCap by a unique, trial-specific identification code. To ensure data quality, regular data audits will be conducted by the Trial Management Group to identify and correct inconsistencies. Staff responsible for data entry will undergo training to standardise procedures and minimise errors. These procedures adhere to Good Clinical Practice (GCP) standards and ensure the reliability of all data collected during the study.

No identifying personal information will be stored alongside the trial data to ensure participant confidentiality. Participants will be allocated a trial-specific identification code and will only be referred to by the code on all research documentation. For paper-based questionnaires returned to the Trial Management Group (TMG), the data will be manually entered into REDCap by research staff. During the study, all electronic data will be securely stored in REDCap. The Google Drive folder will be double password protected and accessible only to authorised members of the research team. Paper copies of questionnaires and informed consent forms will be stored in a locked filing cabinet in a secured office at Oxford Brookes University. Audio recordings of participant interviews will be downloaded immediately after recording and saved as MP3/MP4 files on the Oxford Brookes Google Drive along with the transcriptions of these interviews. Once transcripts have been checked for accuracy, the recordings will be deleted from the recording devices and Google Drive. At the end of the study, all research data will be archived on the Oxford Brookes approved long-term data repository, Arkivum. This data set will not be opened, and data will be stored for a period of 10 years, after which it will be automatically deleted.

### Outcome measures

The primary and secondary outcome measures for this study are designed to evaluate the feasibility, acceptability, retention, and preliminary effectiveness of an HCP-supported self-management intervention for fatigue after critical illness. The outcomes will inform the design and implementation of a future large-scale trial. Furthermore, fidelity and acceptability of the intervention are measured by assessing adherence, estimated frequency, and patterns of use.

#### Primary outcome measures

The primary outcome focuses on the willingness of people affected by fatigue after critical illness to participate in an RCT evaluating an HCP-supported, self-management intervention. Recruitment and retention rates are critical indicators.

Recruitment rate is defined as the number of participants recruited or randomised as a proportion of those eligible and approached. This rate will be assessed at baseline and calculated both overall and by the research site.

Retention rate is defined as the proportion of participants who complete all the required questionnaires and semi-structured interviews. This outcome is essential to evaluate participant engagement and the feasibility of collecting follow-up data.

Assessments will be completed via phone or video call at baseline, three months, and six months post-randomisation. During the baseline data collection phase, additional demographic information (including age, sex, ethnicity, employment status, and marital status) and medical history (including comorbidities, primary diagnosis on ICU admission, ICU length of stay, hospital length of stay, days of mechanical ventilation, and severity of illness assessed by the APACHE III [22] will also be collected.

The progression criteria for full trial are summarised in Table 1.

**Table 1:** Progression criteria

Category	Recruitment	Retention and follow-up completion	Remedial action
Green (Go)	> 85% anticipated (> 60 participants)	At least 80% of survivors will provide complete follow-up data	Continue—no action needed. Apply for further funding
Amber (Amend)	50–85% anticipated (36–75 participants)	60–80% of survivors will provide complete follow-up data	Continue—action needed. Explore ways to improve recruitment/retention
Red (Stop)	< 50% anticipated (< 35 participants)	< 60% of survivors will complete follow-up data	Stop. Explore reasons for poor recruitment/retention

#### Secondary outcome measures

The secondary outcomes aim to estimate retention and the variability of outcome measures and evaluate the acceptability and fidelity of the intervention.

To estimate retention and the variability of outcome measures to inform sample size calculations for a future effectiveness study, completion rates of outcome questionnaires at baseline, three months, and six months post-randomisation will be assessed. These questionnaires will provide comprehensive data on participants’ fatigue levels, coping strategies, functional impairment, symptom responses, and health-related quality of life. Additionally, a comparison between the intervention group and the control group in terms of completion rates and results of questionnaires will be conducted to investigate whether the intervention creates an impact that differentiates the two groups. The questionnaires include the following:

- Carver’s Brief Coping Orientation to Problems Experienced (Brief-COPE) inventory [9]
  The Brief COPE consists of 14 subscales with 2 items per subscale, and categories into 3 dimensions by Cooper et al. [13]: Emotion-focused coping includes acceptance, emotional support, humour, positive reframing, and religion. Problem-focused strategies consist of active coping, planning, and instrumental support. Dysfunctional strategies encompass denial, self-distraction, substance abuse, behavioural disengagement, self-blame, and venting. Each item is rated on a 4-point Likert scale ranging from 1 (“I haven’t been doing this at all”) to 4 (“I’ve been doing this a lot”). It assesses a range of coping responses, including problem-focused coping, emotion-focused coping, and avoidant strategies. Question 6, 8, 11, 13, 16, and 26 are reverse scoring.
- EuroQoL-5 Dimensions 5 Levels (EQ-5D-5L) [19]
  The EQ-5D-5L is a standardised instrument assessing five dimensions of health: mobility, self-care, usual activities, pain/discomfort, and anxiety/depression. Each item is rated on a 5-level scale from 1 (no problems) to 5 (extreme problems). Utility scores will be derived using the van Hout et al. [36] crosswalk mapping function. A visual analogue scale (0–100) captures overall self-rated health.
- Functional Assessment of Chronic Illness Therapy- Fatigue (FACIT-F) [37]
  The FACIT-F is a questionnaire covering five domains: physical well-being, social/family well-being, emotional well-being, functional well-being, and fatigue. Items are scored on a 5-point Likert scale from 0 (“not at all”) to 4 (“very much”). Subscale scores are summed to produce a total score indicating the degree of fatigue and its impact on daily life. Questions GSs, GFs, An5, An7 are reverse scoring.
- Prototype F-ACT Scale (adapted version of Inflammatory Bowel Disease- Fatigue Scale) [15]
  This adapted measure includes: Section I: Fatigue severity (5 items scored 0–4); Section II: Impact on daily activities (25+ items, scored 0–4 with N/A option for question 3, 4, 9, 12, 13, 14); Section III: Additional qualitative questions It captures causes of fatigue, assistance to ease fatigue, as well as duration and frequency of fatigue on physical, cognitive, emotional, and social aspects.
- Work and Social Adjustment Scale (WSAS) [27]
  The WSAS is a 5-item scale measuring functional impairment in work, home management, social and private leisure, and relationships. Each item is scored from 0 (“not at all impaired”) to 8 (“very severely impaired”). Higher scores indicate greater functional impairment.
- Cognitive and Behavioural Responses to Symptoms Questionnaire (Short version) (CBRQ) [30]
  The short version of the CBRQ consists of 18 items across six subscales: Fear avoidance, Damage beliefs, Embarrassment avoidance, Symptom focusing, All-or-nothing behaviour, Resting behaviour. Each item is rated on a 5-point Likert scale from 0 (“strongly disagree”) to 4 (“strongly agree”). Questions FA2 and FA9 are reverse scoring.

Additionally, to evaluate the acceptability and fidelity of the intervention will be evaluated using a combination of quantitative and qualitative methods six months after randomisation. Participants will complete the Theoretical Framework for Acceptability Questionnaire (TFAQ) [32] and the System Usability Scale (SUS) [5] to assess their perceptions of the intervention and the usability of the digital platform.

- Theoretical Framework for Acceptability Questionnaire (TFAQ) [32]
  TFAQ Comprises: Section 1 (All participants) including 6 items covering affective attitude, burden, ethicality, self-efficacy, opportunity costs, and general acceptability and Section 2 (Intervention group only) including 8 items covering the same plus perceived effectiveness and intervention coherence Responses are on a 5-point Likert scale from 1 to 5.
- System Usability Scale (SUS) [5]
  The SUS contains 10 items measuring system usability. Responses range from 1 (“strongly disagree”) to 5 (“strongly agree”). Items alternate between positively and negatively worded statements. Question 2, 4, 6, 8, and 10 are reverse scoring.
- Digital analytics, including the completion of sections from the intervention website will be used to measure patterns of engagement with the intervention components. These data will be collected at the 6-month follow-up and will be included in the evaluation of compliance level.

To be more specific, participants in the intervention group will be evaluated for compliance based on four categories: high compliance, moderate compliance, limited compliance and non-compliance, as summarised in Table 2. Highly compliant participants are those who complete at least 10 sections (66.66% or more) of the intervention, receive the supportive phone call, and set a personal goal with an action plan. Moderately compliant participants complete between 6 and 9 sections of the intervention and fulfil at least one secondary requirement, such as receiving the supportive phone call or setting a personal goal with or without an action plan. Limited compliant participants complete fewer than 5 sections (33.33% or less) but meet at least one secondary requirement. Participants who do not satisfy the criteria for a given category will be classified in the next lower level of compliance, with non-compliant participants being those who fail to meet the criteria for limited compliance.

**Table 2:** Details for compliance levels

High compliance	Moderate compliance	Limited compliance	Non-compliance
**Mandatory requirement**			
Participant has accessed and completed 10 (66.66%) or more sections from the intervention (including goal setting and action planning section)	Participant has accessed and completed more than 5 but less than 10 of the sections from the intervention (including goal setting and action planning section)	Participant has accessed and completed fewer than 5 (33.33%) of the sections in the intervention **or** has not completed the goal and action planning section	Participant does not fulfil the necessary conditions to be considered as **Limited compliant**
**Secondary requirements**			
**(Need to satisfy both or assessed as moderately compliant)**	**(Need to satisfy at least one or assessed as limited compliance)**	**(Need to satisfy at least one or assessed as non-compliant)**	
Participant has received the supportive phone call	Participant has received the supportive phone call	Participant has received the supportive phone call	Participant has not received the supportive phone call
Participant has set a personal goal and devised an action plan	Participant has set a personal goal (with or without an action plan)	Participant has set a personal goal (with or without an action plan)	Participant has not set a goal or an action plan

In addition to quantitative measures, the acceptability of the intervention will be assessed through semi-structured interviews with participants to assess their adherence and provide further understanding of feasibility in the sixth month. Semi-structured interviews of health professionals are also included to evaluate HCPs’ perceptions and experiences of delivering support in this intervention. Topics will include affectivity, burden, ethicality, perceived effectiveness, intervention coherence, self-efficacy, opportunity costs, and general acceptability. These interviews will use a topic guide based on the theoretical constructs of acceptability [31].

### Data analysis

#### Quantitative

Assessments will be performed at baseline, three months, and six months post-randomisation to evaluate feasibility and acceptability. Descriptive statistics will be employed to summarise the baseline demographic and clinical characteristics of both groups. Categorical variables will be reported as frequencies, percentages, and 95% confidence intervals (CI). Continuous variables that are approximately normally distributed will be summarised as means with standard deviations (SD) and 95% CIs. Continuous variables that are non-normally distributed will be summarised using medians with interquartile ranges (IQR).

Feasibility outcomes (including recruitment and retention) will be analysed using descriptive statistics only, consistent with feasibility study methodology. These outcomes will be compared against the pre-specified progression criteria to determine whether they are practical and appropriate to proceed to a definitive RCT.

An analysis of data in the questionnaires will explore potential differences between the groups and whether there is a difference in the change between the groups before and after the intervention to assess the effectiveness of the intervention in reducing fatigue and improving overall quality of life.^1^ Analyses will be conducted based on intention-to-treat (ITT). As this is a small study, treatment effects are likely to have wide CIs and consequently inferences will be tentative and reported as such. Results will be reported in accordance with CONSORT extension to randomised pilot and feasibility trials [16]. A summary of outcome measures, data types, and analysis is listed in Table 3.

**Table 3:**
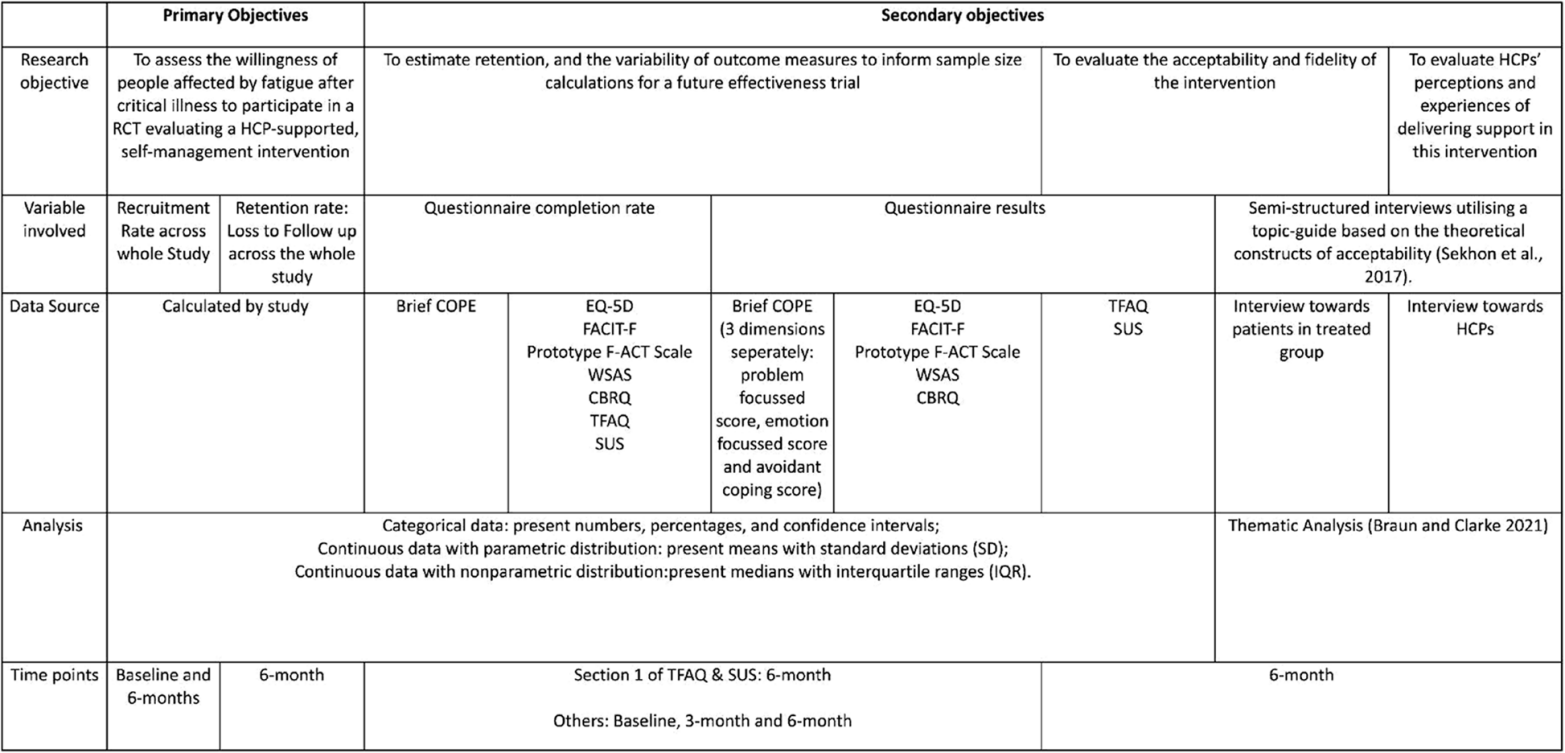
Summary of data analysis plan

#### Qualitative

Qualitative data will be collected through semi-structured interviews, guided by a topic guide developed based on the theoretical constructs of acceptability [31]. These interviews will allow for an in-depth exploration of participants’ perceptions, experiences, and views regarding the intervention. The topic guide will ensure that key areas of interest are systematically addressed while allowing flexibility to follow up on emerging themes during the interviews.

The collected data will be analysed using the six-phase approach to thematic analysis as outlined by Braun and Clarke [4]. This method involves familiarisation with the data, generating initial codes, searching for themes, reviewing themes, defining and naming themes, and producing a final report. This rigorous and systematic approach will ensure that the analysis is comprehensive and provides a nuanced understanding of the qualitative data.

## Monitoring

### Patient and public involvement

The patient advisory group (PAG) consists of the patient co-applicants who will act as chair, the Patient and Public Involvement and Engagement (PPIE) lead researchers, and other patient and carer representatives. This group will meet alternate monthly for the duration of the trial. With support from the PPIE lead, the PAG will review recruitment materials, comment on data analysis, and advise on dissemination materials and strategies, comment on guidance arising from the study and assist in the completion of an impact log.

The PAG group includes people with fatigue after critical illness and HCPs working in critical care rehabilitation have been involved in the development of the intervention and planning of this trial. As part of an earlier study, seven people with fatigue after critical illness, one carer/family member, and seven HCPs co-produced the intervention, and four people with fatigue after critical illness used the draft intervention as part of user testing [7]. Six people with fatigue after critical illness and 4 HCPs have provided input into the development of the trial design and funding submission and reviewed trial documentation, including participant information sheets and invitations. The PPI group will continue to act in an advisory capacity throughout the trial.

### Administrative structures

The study will be monitored by the TMG, which includes the Chief Investigator, Principal Investigators (PIs), patient co-applicant, research team, and the trials unit team. The TMG will meet biweekly during the planning and set-up phases of the trial and every four weeks during the trial’s operational phase. The TMG will ensure adherence to agreed milestones and provide rapid responses to any issues that arise.

In addition to the TMG, the PAG and Clinical Advisory Group (CAG) will provide additional support to the trial. The PAG, chaired by patient co-applicants and supported by PPIE lead researchers and other patient and carer representatives, will meet every two months. Their role will include advising on recruitment materials, reviewing study outputs, contributing to dissemination plans, and supporting the documentation of the study’s impact. The CAG, composed of HCPs who work with patients recovering from critical illness, will meet quarterly to offer input on recruitment strategies, monitor study progress, and guide analysis, dissemination, and implementation processes.

These structured oversight mechanisms will ensure the trial’s progress is carefully monitored, with input from diverse stakeholders, including researchers, clinicians, and patient representatives.

## Ethics and dissemination

### Research ethics approval

Approval for this protocol was granted by the Health Research Authority (HRA) and the NHS Research Ethics Committee (REC) (IRAS ID 328471) on 5th December 2024. Any substantial amendments to the approved protocol or related documents will be submitted for further review and approval by the HRA, REC, and communicated to participating organisations and participants as required.

### Dissemination policy

The findings of this study will be disseminated through diverse channels to effectively reach various audiences. Academic and professional audiences will be engaged through peer-reviewed journal publications, conference presentations, and reports shared with professional organisations and NHS Trusts. Patients and advocacy groups will receive visual abstracts and summaries designed for accessibility, while the public will be reached via social media campaigns, podcasts, and university outreach. Policymakers will receive targeted reports to advocate for evidence-based interventions.

## Discussion

Fatigue after critical illness is a significant and under-recognised problem that profoundly impacts the recovery and quality of life of ICU survivors. Brown et al. [6] and Hosey et al. [20] highlight the urgent need for targeted interventions to address the pervasive and debilitating effects of post-ICU fatigue, emphasising the importance of bridging gaps in post-critical care rehabilitation services. Despite its prevalence, effective strategies to manage fatigue remain limited, underscoring the necessity of innovative, patient-centred approaches.

Findings from this feasibility study will inform the design of the full-scale randomised controlled trial by identifying the optimal conditions for both participant engagement and implementation. Specifically, the study will assess patients’ willingness to participate and determine the most appropriate timing for recruitment, including when survivors feel physically and emotionally ready, have rebuilt trust in healthcare interactions, and are less likely to be overburdened by other research commitments. It will also explore the capacity of research delivery nurses and HCPs at each local site, enabling optimisation of operational processes to ensure smooth collaboration and consistent delivery. Addressing these factors will be essential to enhance recruitment efficiency, support participant adherence, and ensure high-quality intervention delivery in the larger-scale trial.

## Supplementary Information

Additional file 1.

## Data Availability

Any requests for data can be made to the corresponding author who will review these on a case-by-case basis with the study team. A data-sharing agreement would be required in this instance.

## Abbreviations

ANCOVA: Analysis of covariance
ADL: Activities of daily living
CAG: Clinical Advisory Group
CBRQ: Cognitive and Behavioural Responses to Symptoms Questionnaire (Short version)
CI: Confidence interval
CRF: Case Report Form
COPE: Carver’s Brief Coping Orientation to Problems Experienced
EQ-5D: EuroQol-5 Dimensions
FACIT-F: Functional Assessment of Chronic Illness Therapy—Fatigue
FACT: Fatigue after Critical Illness
GCP: Good Clinical Practice
GP: General Practitioner
GSTT: Guy’s and St. Thomas’ NHS Foundation Trust
HCP: Healthcare Professional
HRQoL: Health-Related Quality of Life
HRA: Health Research Authority
ICF: Informed consent form
ICU: Intensive care unit
ITT: Intention-to-treat
NHS: National Health Service
OUHFT: Oxford University Hospital Foundation Trust
PAG: Patient Advisory Group
PPIE: Patient and Public Involvement and Engagement
PI: Principal Investigator
PIL: Participant/Patient Information Leaflet
R&D: NHS Trust Research and Development Department
RCT: Randomised controlled trial
REDCAP: Research Electronic Data Capture
SOP: Standard operating procedure
SUS: System Usability Scale
TFAQ: Theoretical Framework for Acceptability Questionnaire
TMG: Trial Management Group
UHCWT: Warwickshire NHS Trust
WSAS: Work and Social Adjustment Scale

## Footnotes

1 All the questionnaires in the Section 3.8 will be included except for Section 1 of TFAQ and SUS because only the treated group will provide responses. For the Brief COPE, scores across the 3 domains—problem focussed score, emotion focussed score and avoidant coping score, will be analysed separately.
